# Dimensionality Reduction Based Optimization Algorithm for Sparse 3-D Image Reconstruction in Diffuse Optical Tomography

**DOI:** 10.1038/srep22242

**Published:** 2016-03-04

**Authors:** Tanmoy Bhowmik, Hanli Liu, Zhou Ye, Soontorn Oraintara

**Affiliations:** 1 Department of Electrical Engineering, University of Texas at Arlington, Arlington, TX 76019, USA; 2 Department of Bioengineering, University of Texas at Arlington, Arlington, TX 76019, USA; 3 Department of Biomedical Engineering, Mahidol University, Salaya, Nakhon Pathom 73170, Thailand

## Abstract

Diffuse optical tomography (DOT) is a relatively low cost and portable imaging modality for reconstruction of optical properties in a highly scattering medium, such as human tissue. The inverse problem in DOT is highly ill-posed, making reconstruction of high-quality image a critical challenge. Because of the nature of sparsity in DOT, sparsity regularization has been utilized to achieve high-quality DOT reconstruction. However, conventional approaches using sparse optimization are computationally expensive and have no selection criteria to optimize the regularization parameter. In this paper, a novel algorithm, Dimensionality Reduction based Optimization for DOT (DRO-DOT), is proposed. It reduces the dimensionality of the inverse DOT problem by reducing the number of unknowns in two steps and thereby makes the overall process fast. First, it constructs a low resolution voxel basis based on the sensing-matrix properties to find an image support. Second, it reconstructs the sparse image inside this support. To compensate for the reduced sensitivity with increasing depth, depth compensation is incorporated in DRO-DOT. An efficient method to optimally select the regularization parameter is proposed for obtaining a high-quality DOT image. DRO-DOT is also able to reconstruct high-resolution images even with a limited number of optodes in a spatially limited imaging set-up.

DOT reconstructs optical properties of a highly scattering medium in the near infra red (NIR) domain from the measurement of scattered and attenuated optical flux at the surface of the imaging volume. As the absorption of light in human tissues is low in the NIR region, NIR photons can penetrate several centimeters inside the tissue^1^. This makes DOT a promising tool in several biomedical imaging applications such as for brain imaging, breast and prostate cancer detection and molecular imaging^2^,^3^,^4^.

The inverse problem in DOT is highly challenging because of the coupling between unknown absorption and scattering. At the same time, due to the diffusive nature of light propagation and a limited number of measurements, the problem is severely ill-posed and underdetermined^5^,^6^,^7^,^8^. The two most commonly used linearization approaches are Born and Rytov approximation that linearizes the scattered flux and scattered phase respectively with respect to the absorption variation^9^. Assuming that there are *m* sources and *q* detectors accounting for total *m* × *q* measurements and the full 3-D imaging volume is discretized into *n* voxels, the linearized forward model for DOT becomes:


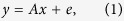


where *y* = [*y*(*d*_1_, *s*_1_), …, *y*(*d*_*m*_, *s*_*q*_)]^*T*^ is the measurement vector, *x* = [*O*(*r*_1_), *O*(*r*_2_), …, *O*(*r*_*n*_)]^*T*^ is the image vector representing the perturbation in absorption coefficient, *e* is the measurement noise and *A* is the sensing matrix given by:


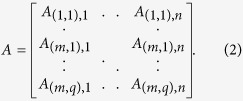


Here *y* ∈ *R*^*mq*×1^, *x* ∈ *R*^*n*×1^, *e* ∈ *R*^*mq*×1^ and *A* ∈ *R*^*mq*×*n*^. The elements of *A* determine the weight of each voxel in every measurement. Hence the sensitivity of the *k*th voxel in the measurement at the detector *d*_*i*_ due to the source *s*_*j*_ is given by *A*_(*i*,*j*),*k*_.

In general equation (1) is underdetermined (since *m* × *q* ≪ *n*) and *A* is ill-conditioned^5^. Thus the DOT linearized inverse problem of determining *x* from *y* is non-unique and unstable. This ill-posedness can be overcome by regularizing the solution using prior information about the image. The most common choice is the Tikhonov-type regularization, where the least-square residual is regularized using the 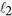 norm of the unknown image defined as 
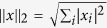
10:


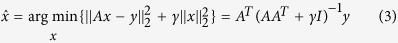


*A*^*T*^ denotes the transpose of *A*, and *γ* is the regularization parameter that controls the balance between the data fidelity and regularization terms. Methods of selecting this parameter for DOT have been well studied^11^. The Tikhonov method is simple and easy to implement, and therefore was widely used in DOT^12^,^13^. 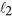 regularized solution can be obtained in real time while suppressing high frequency noises. But the major drawback of Tikhonov approach is poor spatial resolution as the reconstructed image is over-smoothed or blurred^14^. Hence, it is difficult to reconstruct images which are sparse or have distinct boundary with respect to the background.

In many of DOT applications, it is common that the absorption coefficient perturbation is localized occupying only a small portion of the whole field of view^15^. This in turn implies that *x* is spatially sparse and the nonzero voxels exist only around the same neighborhood. Therefore, sparsity based regularization techniques that restrict the number of nonzero elements in the solution warrant attention. The problem of sparse signal recovery is well studied in the area of compressed sensing^16^,^17^,^18^. In practice with respect to DOT, sparseness of the optical heterogeneity is imposed by using the 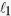-norm regularization for solving the optimization problem^19^:


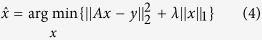


With correct choice of *λ*, the 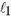 regularized solution 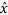 of equation 4 can accurately localize and quantify the absorption anomaly^14^. Researchers in DOT community have successfully used 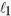 regularization techniques to reconstruct sparse DOT images^7^,^14^,^20^,^21^. However, state of the art 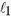 norm minimization techniques involve solving a linear system with the size of the unknown image which ensues a huge computational burden for the full 3-D imaging volume^22^,^23^. Hence the current 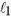-based approach is not suitable or practical for 3-D DOT reconstruction in real time.

A major challenge for the researchers in solving equation 4 is to select the correct regularization parameter. Although for Tikhonov regularization well-researched theoretical guides exist to choose the parameter (*γ*) based on the l-curve and cross-validation method, for 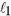 optimization choosing the parameter *λ* is still an unsolved technical problem and the choice is made empirically^24^,^25^,^26^.

The second challenge in DOT is that 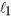 or 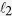 regularized DOT reconstruction suffers from poor depth localization as the sensitivity of DOT measurement decreases exponentially along the depth^12^,^27^. Pogue *et al.* proposed a spatially variant regularization scheme to enhance depth sensitivity^28^. Recently a more direct approach of modifying the sensing matrix to compensate for sensitivity decrease with increasing depth has been adopted^14^,^27^.

Another challenge for DOT research community is the optode geometry optimization, with which high quality reconstruction can be obtained by deploying a minimum number of optodes. Tian *et al.* have studied different optode configurations to find the optimal measurement density using 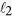-based regularization^13^.

In this paper, we demonstrate a novel reconstruction method that is able to overcome the challenges mentioned so far for solving the sparse DOT inverse problem. For sparse images, the size of the linear system for the inverse problem can be reduced drastically if one can approximately create a low-resolution support mask of the nonzero voxels beforehand. Motivated by this insight, we have developed a two-step, dimensionality-reduction-based optimization (DRO) algorithm for DOT image reconstruction. In the first step, DRO-DOT finds a low resolution support mask with potential nonzero voxels by identifying and grouping the sensing columns in *A* and the corresponding voxels of *x*. The number of such groups is far fewer than the number of original voxels. In the second and final step, 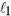 minimization is carried inside the recovered support mask only, whose size is smaller than the full 3-D imaging volume. Hence in both steps the number of unknowns is reduced, resulting from dimensionality reduction of the original problem and thereby scaling down the computational complexity. In addition, the sensing matrix *A* is re-weighted to enhance the depth sensitivity of the overall method by including depth compensation. The critical problem of chosing the regulariation parameter was also addressed by developing an adaptive scheme to find *λ* based on the statistical interpretation of 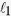 regularization in equation 4. By the end of this paper, we will show that DRO-DOT is able to recover high-resolution images even with a limited number of optodes, which in turn reveals the possibility and feasibility of using transcretal DOT for prostate cancer imaging.

Overall, the major novelty of our approach is to solve an optimization model that consists of a data residue item and a sparse regularization item. The algorithm leads to three advantages: (1) It forms a low resolution supporting basis to reduce computing complexity. (2) It refines the depth compensation algorithm so as to recover more accurate DOT images. (3) It offers a semi-automatic method to choose optimal regularization parameter.

## Methods

### DRO-DOT

Let the original sparse image *x* ∈ *R*^*n*^ satisfies 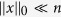 where 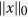 is the 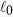 norm of *x* that is equal to the number of nonzero voxels, and *I* is the set of indices of the nonzero voxels, i.e. *I* = {*i* : *x*_*i*_ > 0}. Hence 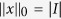. Let *I*′ be a subset of {1, 2, ..., *n*} which contains the nonzero voxel locations i.e., 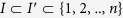. If it is possible to approximate such a support *I*′ so that 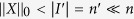, then the original forward problem of (1) can be re-stated as:


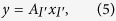


where *x*_*I*′_ and *A*_*I*′_ are the sub-vector and sub-matrix formed within voxels of *x* and columns of *A* chosen from the list *I*′ respectively. Solving (5) is faster than solving the original problem (1), because now the dimensionality of the inverse problem is reduced to *n*′ from *n*. This insight leads us to the first step of DRO-DOT.

#### Step-1

The original high-resolution 3-D voxel space is first transformed into a lower resolution voxel space and then the support of the heterogeneity is found in that space. Remember that *A* is the sensing matrix whose *j*th column corresponds to the measurement sensitivity in all the source-detector pairs for the *j*th voxel. Because of the diffusive nature of light propagation, the measurement sensitivity for *x*_*j*_ and *x*_*k*_ will be highly similar or correlated if these voxels are close. Thus the columns of *A* corresponding to the spatially close voxels should be highly correlated. This important rationale leads to the procedure for forming the low resolution voxel basis as follows: the algorithm starts from the first column of *A* i.e. *A*_1_ and finds all columns that are highly correlated with *A*_1_ (for example, setting a correlation coefficient threshold >0.95). Then these correlated columns are grouped together as 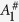. The algorithm repeats the same process from the residual list of columns and continues the grouping process until *A* is fully exhausted. Let *n*^#^ groups are formed and each group is represented by the first column member of that group. The columns representing the *j*th group is denoted by 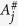. For each group of correlated columns we take the sum of the corresponding elements of *x* in that group (because of one-to-one correspondence between column locations and voxel positions) to form *x*^#^ which is the low resolution image basis of *x* such that 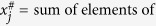 *x* corresponding to *j*th group of voxels. After forming this new basis, equation (1) can be approximated as:


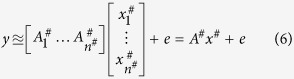


Choosing the correlation threshold is a crucial decision for performing the dimensionality reduction (from *n* to *n*^#^) in step-1. Suppose *τ* to be the correlation threshold. If *τ* is very high, there will be fewer number of columns in each group and hence *n*^#^ will be large and the benefit of dimensionality reduction will be lost. Again, if *τ* is very low, the approximation error 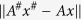 will be high. Hence it is important to find a trade-off between the approximation error and dimensionality reduction. We will choose the effective value of correlation threshold to be *τ*_*e*_, such that the relative approximation error 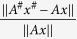 remains less than 5% to ensure that we do not sacrifice accuracy in our pursuit to reduce computational burden. More details on how to select the effective correlation threshold *τ*_*e*_ are given in Section 1 of Supplementary Information.

Note that the collection of nonzero elements of *x*^#^ will correspond to the support for the sparse object only if the nonzero elements of *x* are of the same sign. Otherwise, a scenario is possible where the sum of nonzero voxels with different signs in some *k*th group is zero which will make 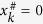. Hence, to recover the support of the sparse object by finding nonzero elements of *x*^#^, it needs to be assured that the nonzero elements of *x* are of same sign, which in turn guarantees that if any *k*th group of voxels from *x* contains any number of nonzero elements, then 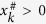. Fortunately, for typical DOT imaging scenarios such as brain activation, presence of tumor etc., the change in absorption coefficient in the region of interest is known to be positive and researchers have used this non-negativity constraint to design their optimization strategies^29^,^30^. With such positivity constraint on *x*, one can find this support by solving the modified version of equation 4 adapted to the new basis:


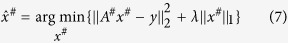


This is the crucial step leading to the success of DRO-DOT. Equation 7 is solved using split augmented lagrangian shrinkage algorithm (SALSA) which is widely used for solving 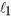-minimization problem^22^. The convergence of SALSA for 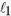 regularization has been proven in section III B of ref. 22. In particular, the algorithm is said to converge when the relative change in the objective function falls below some pre-set tolerance limit. As an example, in the Supplementary Information Section 2, we plot the evolution of the objective function vs time for one of the experiments reported in this paper and visually illustrate the convergence of our algorithm. Also SALSA was initialized with a zero vector, because in general, one can not have prior knowledge of the position, size or contrast of the imaged object or cancer lesion.

#### Step-2

After finding the low resolution voxel basis, this basis is mapped back to the original voxel basis to get *I*′ as discussed before. 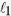 minimization is then performed inside this support *I*′ instead of the full imaging volume. Therefore one needs to solve the new optimization problem associated with equation (5):


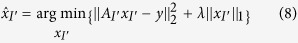


As the number of voxels in *I*′ is *n*′, which is much smaller than *n*, the optimization problem in equation 8 is computationally inexpensive.

### Depth Compensation

As the measurement sensitivity degrades exponentially along the depth of the tissue, reconstructed image becomes biased towards the surface. This problem of poor depth localization was addressed earlier by multiplication of the *A* matrix and a spatially varying regularization approach^27^,^28^. In the current work, the first approach is followed with modification. For *n* = *n*_*x*_ × *n*_*y*_ × *n*_*z*_, *A* can be re-written as concatenation of *n*_*z*_ block matrices corresponding to *n*_*z*_ layers from the measurement surface:


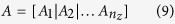


In principle, sensitivity of *A*_*i*_ should be bigger than sensitivity of *A*_*j*_ if *i* < *j*. To equalize or compensate for this sensitivity attenuation along the depth, each block of *A* is reweighted as follows^14^:


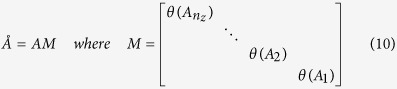


where *θ*(*A*_*i*_) is the maximum singular value of *i*th block or layer. Thus the sensitivity for the last layer is boosted most and for the first layer it is suppressed most. Now one can obtain a depth localized image by solving the modified optimization problem of equation 4 as follows:


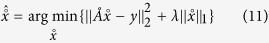


We added one more step to find the final optimized solution 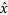 from 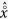 as:


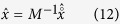


Equation (12) preserves the quantification of the reconstructed image after depth equalization.

### Selection of Regularization Parameter

It is known that the success of 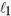 regularization depends on the correct selection of regularization parameter *λ*. To avoid biasing of the reconstruction result, this choice should not be based on a trial-and-error approach^26^; rather, an automatic selection criterion is required. In this research, a semi-automatic method of choosing *λ* is proposed for the standard DOT experimental paradigms. The method is based on statistical interpretation of the regularization parameter as a ratio of measurement noise level and sparsity parameter. It can be shown that the solution of equation 4 is indeed the maximum a posteriori (MAP) estimator for the linear model (1) with a Laplacian prior and Gaussian noise model, as given below:


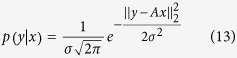



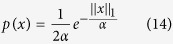


where *σ*^2^ is the measurement noise variance and *α* is the sparsity parameter. The MAP estimator can be found by maximizing the joint probability which results in the following convex optimization:


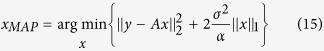


Comparing (15) with equation 4 readily gives:


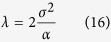


In general, one does neither know the noise variance nor the sparsity of the original image in advance. Hence making the right choice for *λ* requires trying different combination of these two independent parameters and it is not known which one is the right combination. It is possible to have a good estimate of the noise variance *σ*^2^ from an adequate number of DOT measurements, owing to the excellent sampling rate and thus temporal resolution of DOT. Also the range of *α* can be estimated based on clinical/biomedical knowledge and estimation. As an example, for prostate or breast cancer, even though the tumor location, shape and severity are unknown, we would expect only a few suspicious lesions, namely, to have a few sparse objects to be imaged and found. Knowing a realistic range, the sparsity parameter *α* can be estimated. This range can be discretized as {*α*_1_, *α*_2_, …*α*_*l*_}, where *α*_1_ and *α*_*l*_ correspond to the minimum and maximum possible value of the sparsity parameter. This gives a range of *λ* values where 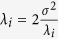. The appropriate *λ* can be chosen from that range based on the discrepancy principle^31^. According to the discrepancy principle the correct choice of *λ* will make the data discrepancy equal to the noise variance. Hence the optimum *λ* denoted by 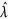 is chosen such that,


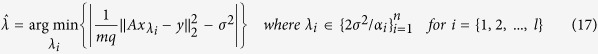


where 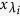 is the solution for 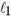 regularization with *λ* = *λ*_*i*_. The practical utility of this method is of utmost importance to reconstruct tissue properties in real time without any assistance from other imaging modalities.

### Evaluation of the Reconstructed Image Quality

Four parameters were used to evaluate the quality of reconstruction with DRO-DOT:

#### Area Ratio (AR)

This quality metric is defined as the area ratio between the reconstructed object and original object for 2D images^13^:


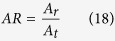


where *A*_*r*_ and *A*_*t*_ is the area of reconstructed and true object in 2D, respectively. *A*_*r*_ is calculated using the full width half maximum (FWHM) approach.

#### Volume Ratio (VR)

For 3D reconstruction, a more relevant evaluation metric is volume ratio (VR), as introduced:


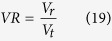


where *V*_*r*_ and *V*_*t*_ is the volume of reconstructed and true object in 3D, respectively. *V*_*r*_ is also calculated using FWHM in 3 dimensions.

#### Contrast Ratio (CR)

We define CR as the ratio of the mean value of reconstruction in the region of interest(ROI) i.e., inside the true object boundary and mean value of the background (BG) outside the object boundary, as given by:


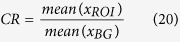


As *x* represents the change in absorption coefficient, ideally *x* = 0 in the background and hence for perfect reconstruction, *CR* → ∞.

#### Run Time (RT)

RT is the total time taken by the reconstruction algorithm to complete the computation process, assuming the regularization parameter is already selected.

## Results

### Performance Evaluation of DRO-DOT

**Figure 1:**
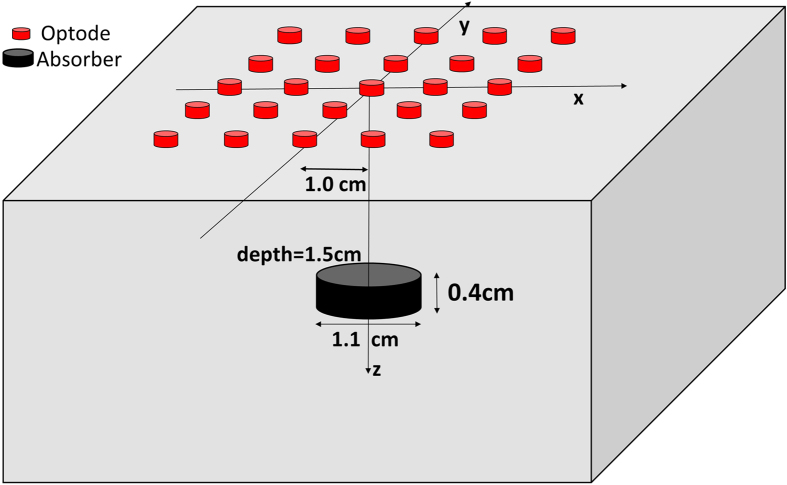
Schematic diagram of the experimental setup. The optode array is a 5 × 5 geometry of bifurcated source-detector optodes with a 1 cm separation between neighboring optodes. A cylindrical absorption anomaly is placed into the intralipid phantom along the center of the grid at a depth of 1.5 cm below the surface.

The accuracy of newly developed DRO-DOT algorithm has to be evaluated in terms of localization and quantification of reconstructed objects. Also the computational efficiency needs to be independently evaluated. To establish or demonstrate the superiority of DRO-DOT, we compare DRO-DOT with two other state of the art optimization techniques extensively used for DOT reconstruction. The first one is Tikhonov regularization and second one is the conventional 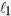 regularization used for sparse object recovery. The experimental set-up is illustrated in Fig. 1. This laboratory phantom experiment was designed to mimic the brain imaging paradigm, where 5 × 5 optodes with a minimum optode separation of 1 cm were arranged on the surface. The 25 optodes were bifurcated, permitting each of them to transmit and detect the NIR light through the tissue phantom. A black cylindrical disk of 1.1 cm in diameter and 0.4 cm in thickness was placed into the 1% intralipid phantom, with one circular side facing up. The depth of the center of the absorbing object was 1.5 cm and it was located along the center of the *x* − *y* plane. For more details about the set-up and instrumentation, the reader can refer to the work of Tian *et al.*^13^.

As described in the Method step-1 section, we plotted the relative approximation error 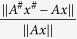 for different values of correlation threshold and observed that the relative error goes below 5% for *τ* ≥ 0.96. Hence we set *τ*_*e*_ = 0.96 which reduces the dimensionality by more than 80% in step-1. The tolerance limit for convergence of the 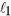 regularized optimization was set to be 10^−5^, namely, the calculation stops when the relative difference in the cost function becomes lower than 10^−5^. We found *λ* = 0.0251 by applying our regularization parameter selection approach. For further details about the variables and parameter settings, the intereseted reader can refer to Supplementary Information Section 3.

**Figure 2:**
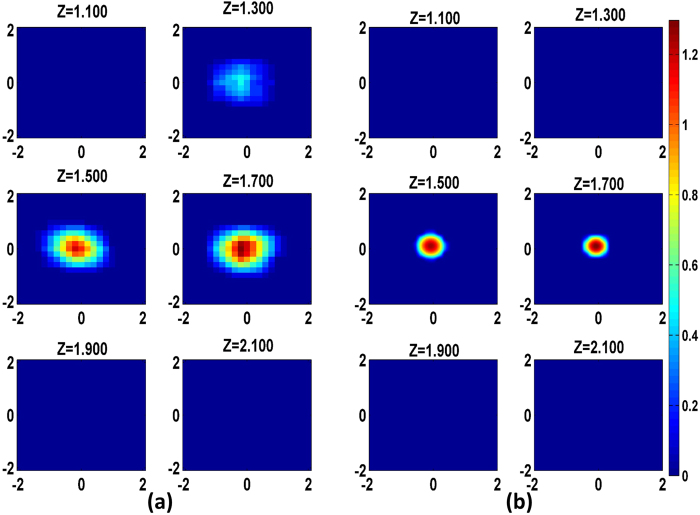
DRO-DOT step by step reconstruction for the tissue phantom of Fig. 1. (**a**) The low resolution image support region recovered in Step-1 and (**b**) The final reconstructed image obtained in Step-2 by solving 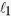 optimization inside the support region obtained in Step-1. The X axis, Y axis, and z are all in cm. The color bar represents the absorption coefficient.

Figure 2 depicts the two-step reconstruction of the object at sequential layers for the phantom experiment shown in Fig. 1. It is known that the sensitivity pattern for each source-detector pair follows a banana shape, and outside of that shape objects are not detectable^32^. Hence the imaging volume for reconstruction was taken to be 4 *cm* × 4 *cm* × 2.5 *cm*. Initial visual inspection of the reconstruction clearly shows that DRO-DOT recovers the image location in 3D and size with good accuracy and high contrast [Fig. 2]. And it is important to note that owing to the novel regularization parameter selection approach, the reconstruction was done without any prior information on the true size and location of the object. At the same time the computational complexity was greatly reduced: Step-1 took 1.9 s and Step-2 took just 0.17 s to achieve convergence. Given that this was 3D reconstruction, the time taken was much shorter than conventional 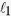 based methods. This shows the promise-of DRO-DOT for real time functional DOT imaging applications. At this point, it is warranted to have a fair comparison between DRO-DOT and other state of the art DOT recovery algorithms.

**Figure 3:**
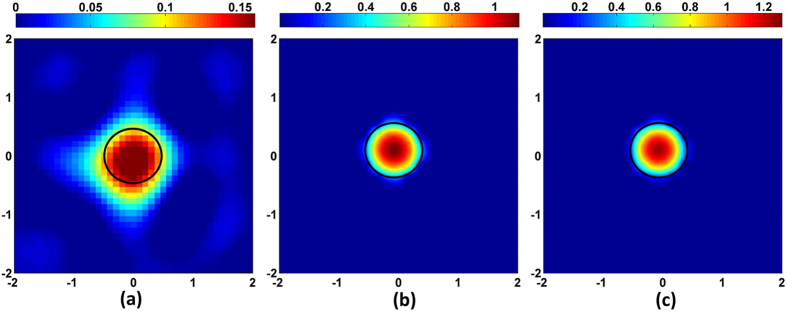
Reconstruction at Z = 1.5 cm plane using (**a**) 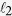-norm minimization method (**b**) 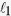-norm minimization method and (**c**) DRO-DOT. The black circle represents the perimeter of the true object in each case. The X axis and Y axis are all in cm. Color bars represent the absorption coefficient.

DRO-DOT is compared to two other conventional methods that are extensively used for DOT reconstruction: 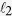 norm and 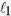 norm based optimization respectively. Figure 3 shows the image reconstruction at a plane of depth 1.5 cm from the surface (along the centre of the object). As can be seen, both DRO-DOT and 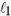 based method outperform 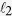 minimization approach in terms of image localization and quantization. 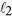 minimization is fast, easy to solve and has the automated regularization parameter selection such as *l* − *curve* and Generalized Cross Validation method^26^,^31^. But it suffers from over-smoothing effect as shown in Fig. 3a. 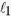 minimization promotes sparseness and hence good quality reconstruction, but the computational burden is very high for a large number of voxels which is indeed a challenge for 3D DOT reconstruction. Also the choice of regularization parameter in 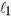 based approach is heuristic which needs prior information of image size and location. We claim that our method gives at least as good quality as 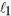 minimization while using a small amount of computational time without any prior information to find an optimal regularization parameter.

At this point, a quantitative comparison between DRO-DOT and the state of the art techniques should be carried out in terms of different quality metrics following the common practice^13^,^14^,^33^. Table 1 compares the performance of DRO-DOT with other two optimization schemes in terms of reconstruction quality and speed. It shows that *AR* and *VR* values are close to unity by DRO-DOT and pure 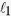 method, whereas these values are greater than 1 by 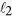 regularization because of the over-smoothing effect. DRO-DOT and the 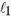 method also offer approximately 4 times better contrasts than the 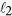 method. The run time of DRO-DOT is as short as that of the 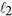 method and 5 times faster than the pure 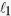 method. Thus DRO-DOT achieves best of the both aspects: enhanced quality of 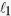 reconstruction and high speed of 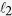 optimization.

### Depth Compensation

**Figure 4:**
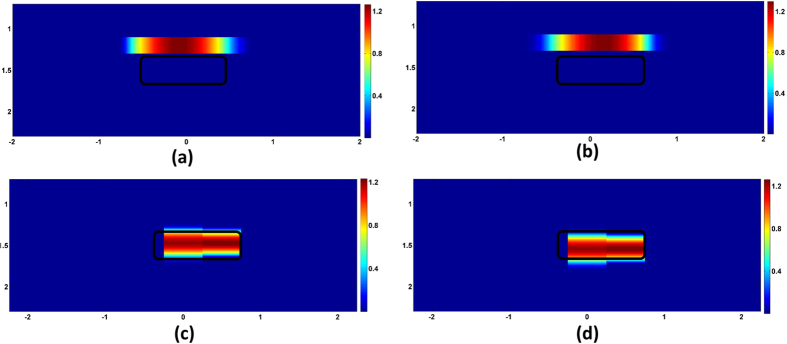
Reconstruction along the vertical planes with and without DC. The first row shows reconstruction without DC along (**a**) *y* − *z* plane (**b**) *x* − *z* plane. Reconstruction with DC is in the second row (**c**) *y* − *z* plane (**d**) *x* − *z* plane. The original object outline is shown in black. Dimension units for each panel are in cm. Color bars represent the absorption coefficient.

The sensitivity of the measurement wanes rapidly with increasing depth. Hence the reconstruction is bound to be biased near the surface. By incorporating depth compensation (DC) in DRO-DOT, it is possible to reconstruct a DOT image at correct depth. Figure 4 shows how depth compensation mitigates the depth-biasing effect. The reconstruction with and without depth compensation is shown along the two vertical planes *y* − *z* and *x* − *z*. It can be seen that without DC, the reconstruction comes near the surface and the depth of the object center is wrongly biased at 1.1 cm. With DC the center is reconstructed at 1.6 cm depth which is close to the original (1.5 cm).

### Optode Placement

In Fig. 1, the set up was with a dense array of 25 bifurcated source and detectors. Such a setting can be afforded in cases where a wide open area is available for multiple optode placements such as for brain imaging and breast cancer detection. However, in some clinical applications where a very limited space is available to place an adequate number of optodes, such as transcretal imaging for prostate cancer detection, DOT will face a major challenge in order to achieve high-resolution images^34^. Even in case of functional brain imaging, lengthy set-up time needed for many optodes adds to subject’s discomfort^13^. But decreasing the number of optodes or measurements makes the DOT image reconstruction more ill-posed and thus the reconstruction quality is bound to suffer. Hence it is interesting and important to know how much one can afford to decrease number of optodes without degrading image quality. Such a study had been done earlier by Tian *et al.*^13^, but the reconstruction method used was based on 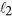 minimization, and hence in general, it suffered from the oversmoothing effect. As DRO-DOT is already 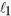 based and therefore promotes sparsity, in general, it is expected to achieve better quality reconstructed DOT images for all different geometries.

**Figure 5:**
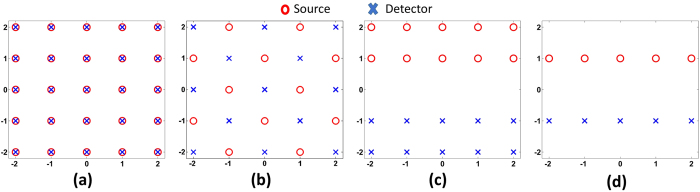
Different optode geometries (**a**) SD-I (**b**) SD-II (**c**) SD-III (**d**) SD-IV.

Figure 5 lists four different source detector geometries to be evaluated for comparison. They are named as geometry SD-I, SD-II, SD-III and SD-IV. SD-I is the same geometry being used in the phantom experiment [Fig. 1] and results in reconstructed DOT images shown in Figs 2, 3, 4. SD-II is also 5 × 5 optode geometry similar to SD-I, with sources and detectors placed alternately (i.e. no bifurcation). SD-III and SD-IV are derived from SD-I by discarding one row and three rows of optodes, respectively, also without bifurcation. For SD-II to SD-IV, the data sets of measurements are selected or reduced from the original measurements in SD-I.

**Figure 6:**
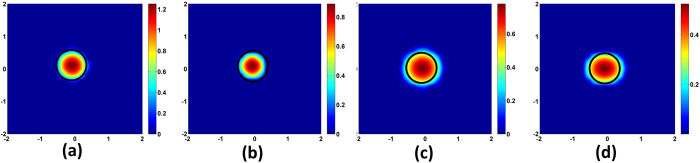
Reconstruction using DRO-DOT at *x* − *y* plane at depth 1.5 cm for (**a**) SD-I (**b**) SD-II (**c**) SD-III (**d**) SD-IV. The X axis and Y axis are all in cm. Color bars represent the absorption coefficient.

Figure 6 shows the reconstruction for the four different optode geometries at the depth of 1.5 cm. It can be seen that the reconstructed image size and location are recovered almost exactly for each case, except for some difference in quantification.

In addition, Table 2 shows that using DRO-DOT, the quality of the reconstruction is maintained high even with a very limited number of sources and detectors used.

### Feasibility of DRO-DOT for Transcretal Prostate Cancer Imaging: *Tissue Phantom Study*

**Figure 7:**
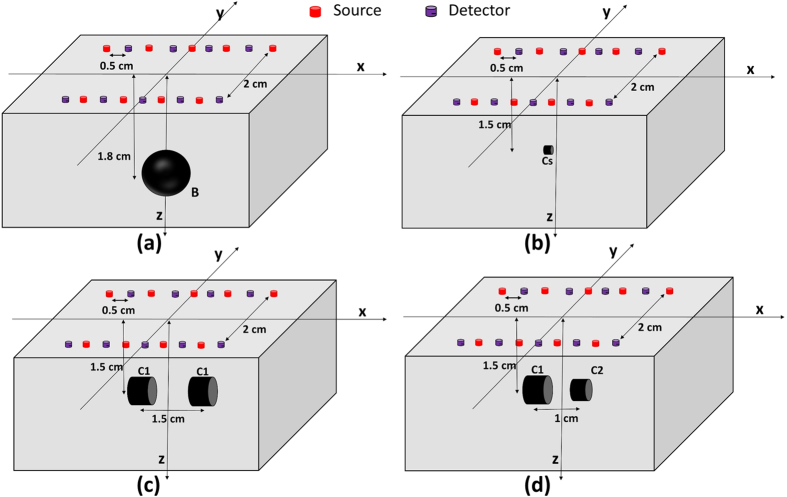
Experimental set-up for different phantom configurations: (**a**) single object of type *B* placed at a depth of 1.8 cm. (**b**) single tiny object of type *Cs* placed at a depth of 1.5 cm, (**c**) dual identical objects of type *C*1 placed at 1.5 cm depth with center to center separation of 1.5 cm, and (**d**) dual objects of two different types *C*1 and *C*2 placed at 1.5 cm depth with center to center separation of 1 cm.

In the previous section, it has been demonstrated that even when there are two rows of optodes, DRO-DOT is able to localize the object at the correct depth and position with a high contrast. This observation leads us to focus on a challenging application of DOT: transrectal prostate cancer imaging. In prostate imaging, the optode probe has to be inserted through the subject’s anus where a very limited space is available for optode placement and only two rows of closely placed optodes can be used^34^. To investigate the feasibility of DRO-DOT for transrectal imaging, several laboratory experiments were carried out with the optode set-up shown in Fig. 7. Nine sources and nine detectors were placed alternately in two rows making a total number of 81 measurements. The two rows were separated by 2 cm and the closest optode separation was 0.5 cm. The homogeneous background was 1% intralipid solution, giving the background absorption coefficient of ≃0.03 *cm*^−1^.

Four different phantom configurations as shown in Fig. 7 with objects of different shapes and sizes were used to evaluate 3D image reconstruction using DRO-DOT. Object *B* was a spherical ball with ≃0.9 cm diameter. *Cs* was a much smaller object of cylindrical shape with a diameter of ≃0.2 cm and a length of ≃0.3 cm. Object *C*1 was a cylinder with a diameter of ≃0.85 cm and a length of ≃0.62 cm. Object *C*2 was also a cylinder but with diameter of ≃0.65 cm and a length of ≃0.45 cm. In the first experiment, *B* was placed at a depth of 1.8 cm (Fig. 7a). The second experiment was more challenging as we tried to reconstruct a tiny object *Cs* that was placed at a depth of 1.5 cm (Fig. 7b). The other two experiments were also interesting as those needed to reconstruct dual objects. In one case, two *C*1 objects were placed at 1.5 cm depth and were separated by 1.5 cm center-to-center distance making the separation between their close surfaces around 0.8–0.9 cm (Fig. 7c). In the fourth experiment, two cylinders with different sizes i.e., *C*1 and *C*2 were closely spaced with a 1 cm center-to-center distance or 0.45 cm surface-to-surface distance at 1.5 cm depth (Fig. 7d). The imaging volume used in DRO-DOT was chosen to be 6 *cm* × 4 *cm* × 2.5 *cm* right below the optode surface.

**Figure 8:**
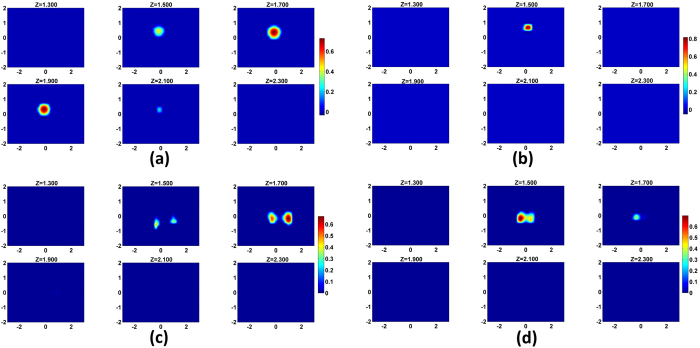
Reconstructed DOT images from four different DOT phantom experiments using transrectal geometry. (**a**) Set-up 1: Object *B* placed at 1.8 cm depth, (**b**) Set-up 2: Object *Cs* placed at 1.5 cm depth, (**c**) Set-up 3: Two identical cylindrical objects of both *C*1 separated by a 1.5 cm center-to-center distance at 1.5 cm depth, and (**d**) Set-up 4: Two different sized objects *C*1 and *C*2 with a 1 cm center-to-center separation at 1.5 cm depth.

Figure 8a shows the reconstructed image using DRO-DOT for the ball-shaped object at different depths. The size and location of the spherical object was recovered accurately. Figure 8b shows the ability of DRO-DOT to reconstruct an object of dimension as low as 0.2 cm. For the first dual-object phantom experiment, two *C*1 objects are resolved with good accuracy as seen in Fig. 8c. For the second dual-object case with one *C*1 and one *C*2 object, the reconstruction is shown in Fig. 8d. In this case, though the two objects are distinguishable, there no more exists a clear boundary between them. This experiment demonstrates that DRO-DOT can not separate objects separated less than 5 mm.

## Discussion

In the past 2 decades, a large amount of research work has been done and published in the area of model-based reconstruction in DOT, as reviewed by the two refs 6, 7. The former one has reviewed about 200 published articles, while the latter one lists 500 references. In addition, several groups have utilized model-based DOT to obtain ultrasound guided tomography, fluorescent tomography and photo-acoustic tomography^35^,^36^,^37^. In comparison, we have developed a novel algorithm to rapidly reconstruct high quality 3D DOT images which can be potentially used in real time in future. The novelty of the algorithm rests on the formation of a low resolution supporting basis in its first step by grouping highly correlated columns within the sensing matrix. This step enables one to solve the inverse problem using 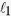-minimization with a small number of low resolution voxels so as to find the approximate image support. Traditionally any prior information from other imaging modalities, such as magnetic resonance imaging(MRI), positron emission tomography(PET) or ultrasound (US) is always useful to provide additional mathematical and/or anatomical constraints that ultimately lead to higher quality reconstructed images^4^,^34^,^38^. In general, however, acquisition of multi-modality images is not always feasible and adds burden on healthcare costs. The first step of DRO-DOT effectively addresses this issue by being able to recover the image support without any prior information. Within this low-resolution support, the true object can be found in a very short time in the second step using 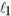 optimization. Thus DRO-DOT algorithm achieves the superior quality of 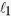 optimization and at the same time remains computationally inexpensive.

The experimental validation of DRO-DOT to examine the quality and speed of image reconstruction has been performed using standard laboratory phantom experiments. Reconstructed images using DRO-DOT were compared with those by state of the art 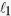 and 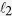 based optimization techniques. The reconstructed image quality is quantified by such metrics as AR, VR, and CR. Also, computational complexity for each algorithm was judged by comparing their respective runtimes. Table 1 shows that both our method and 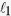 minimization perform excellently leading to high-quality reconstructed images, whereas 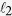 minimization does not offer recovered images with high spatial resolution and contrast. This result is expected because the final step of DRO-DOT actually runs 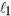 minimization inside the support basis found from the first step, which in principle gives rise to an improved spatial resolution compared to images obtained by 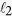 minimization. The total runtime for our method and 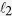 minimization is found to be around 80% less than the runtime of 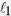 method. The improved computational speed in DRO-DOT stems from the fact that the computational burden of 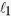 optimization with the huge number of voxels in the 3D imaging volume is reduced by breaking the process into two steps. Both of the steps actually reduce the dimensionality of the original large sensing matrix and hence reduce the computational burden. In this way, DRO-DOT has integrated the optimal aspect of two existing state of the art 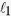 and 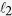 based optimization techniques, i.e., high solution quality of 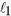 optimization and high speed of 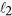 optimization.

The problem of depth localization has also been addressed in the present work. The sensing matrix *A* was modified by assigning more weights to the deeper layers of *A* using equation (10). As shown in Fig. 4, this approach reveals the details in deeper layers and preserves the correct depth in the reconstruction, in contrast to the poor depth localization without depth compensation. We would like to acknowledge that a similar depth compensation method was earlier used by Kavuri *et al.*^14^, though they did not do the correction step as described by equation (12). While this step requires only multiplication of the post-processed image 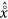 by the inverse diagonal weight matrix *M*^−1^ to reach 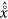 , it is very important for recovering accurate quantification of the imaged object. An earlier study reports that quantification of absorption perturbation can be recovered with a best rate of 64% in simulative experiments for reconsructed images^39^; our current approach with equation (12) permits much improved recovery rate of 99.1%.

As DRO-DOT requires utilization of 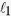 minimization to solve the inverse problem internally, choosing the correct or appropriate regularization parameter *λ* is a major challenge. This challenge is addressed by developing an efficient scheme to choose *λ* based on the statistical interpretation of its dependence on noise variance and the discrepancy principle. This simplification is possible specifically for DOT scenario because *σ*^2^ can be estimated accurately from multiple data collections of the measurements. Then, DRO-DOT is able to systematically select an optimal regularization parameter *λ* without time- and effort- consuming, subjective search of such a parameter.

The study has also clearly demonstrated that DRO-DOT an achieve good-quality DOT image reconstruction even with a limited number of optodes. Figure 6 shows that one can optimize the optode setting geometry, namely, reduce the total number of optodes, without significantly sacrificing the image quality. Although reducing the number of optodes makes the inverse problem (i.e. image reconstruction) more ill-posed and underdetermined, our method compensates for this ill-posedness by reducing the dimensionality of the problem. DRO-DOT is still able to achieve excellent VR and CR (Table 2) after the source-detector pairs are reduced from 25 × 25 to 5 × 5 (Fig. 5). This result is an excellent improvement compared to the previously known results^13^.

Based on the observation that DRO-DOT works well even with limited space to place an adequate number of optodes, its application has been extended to prostate cancer imaging. The real imaging scenario was emulated using laboratory phantom experiments specially designed for this purpose, namely, having a transcretal optode setting (Fig. 7). DRO-DOT is able to reconstruct single and dual objects with accurate localization and high CR. We also learned that the reconstruction algorithm can not separate two small objects if their separation is less than 5 mm. Early research has shown that the spatial resolution in DOT can not be less than 5 mm^2^. Hence our algorithm has achieved the highest possible spatial resolution in DOT. It is worth to compare the current work with the reports by Piao *et al.* and Kavuri *et al.* on prostate DOT studies^4^,^34^. Their methods are based on 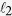 regularization and for 3D imaging, they are computationally expensive as both of the methods uses the NIRFAST software package to do the forward and inverse calculation using the finite element method at each iteration^40^. In contrast, DRO-DOT uses 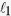 regularization and is very fast or computationally inexpensive.

In reality, biological tissues are often heterogeneous and cannot be rigorously labeled as ‘homogenous’; however, the latter condition is much required in order to mathematically solve model-based inverse problems. Thus, in practice, normal biological tissues are often assumed to be ‘homogenous’ when compared to ‘cancerous’ tissues. Such an assumption has been approximately idealized and utilized in the field of model-based DOT image reconstruction over the last two decades^4^,^6^,^7^. Also, it is well known that DOT’s spatial resolution is limited by the nature of light scattering in tissue. Our new method should actually help improve the spatial resolution and be able to identify a couple of major lesions that are suspicious for cancer.

Although the results by DRO-DOT are promising for DOT imaging with improved resolution and computational speed, we need to recognize a few weaknesses of this study and to possibly improve them in the near future. For DRO-DOT to perform well, the object to be imaged should be both sparse and localized. Such constraint is needed for *x*^#^ to be sparse in the low dimensional voxel space (eq. 6), which in turn guarantees the success of solving equation 7. For example, if the nonzero voxels of x are randomly scattered, then *x*^#^ will have an equal number of nonzero regions in *x*, and hence the dimensionality reduction of the support basis cannot be achieved through step-1. Fortunately, such cases with multi focal lesions are not too common in practice, and most of the time there are not more than two foci for us to focus on^41^. Another limitation is the inability to simultaneously reconstruct absorption and scattering perturbation with the current setup and corresponding algorithm. We used a CW DOT system which measures light intensity and allows only to reconstruct the absorption perturbation assuming a constant scattering background inside the tissue. Although such an assumption of constant scattering coefficient is valid for brain tissue, prostate cancer lesions may have a varied scattering coefficient. A frequency domain (FD) measurement system is needed in order to image both absorption and scattering perturbation using the detected amplitude and phase information of light fluence^7^. In our future work, we will perform experiments with a FD system in order to more accurately image and characterize prostate cancer. Last, the feasibility study of using DRO-DOT for transrectal prostate cancer imaging was carried only on laboratory tissue phantoms, which are quite different from realistic true tissue specimens. It is necessary to conduct either *ex vivo* human prostate specimen measurements or *in vivo* animal experiments to confirm the validity of DRO-DOT for transrectal prostate cancer imaging.

In conclusion, a novel method, DRO-DOT, for 3D DOT reconstruction has been developed and experimentally supported in this paper. It can rapidly reconstruct high-quality images in very short time. Our method does not require prior information from other imaging modalities and thus holds promise for cost reduction and patient convenience, while keeping high quality of image reconstruction. DRO-DOT also incorporates procedures for accurate depth localization and systematic/automatic selection of regularization parameter. As a specific example, several laboratory tissue phantom measurements with transcretal imaging geometry were taken, and the optimal performance of DRO-DOT in respective imaging scenarios has been demonstrated. Further evaluation of our method with animal tissues *ex vivo* and/or *in vivo* as well as with human specimen measurements will enhance and better validate our algorithm. Overall, our method provides a new platform for rapid high-resolution DOT imaging ready for clinical translation in medical imaging applications.

## Additional Information

**How to cite this article**: Bhowmik, T. *et al.* Dimensionality Reduction Based Optimization Algorithm for Sparse 3-D Image Reconstruction in Diffuse Optical Tomography. *Sci. Rep.* **6**, 22242; doi: 10.1038/srep22242 (2016).

## Supplementary Material

Supplementary Information

## Figures and Tables

**Table 1:** Comparison of DRO-DOT with pure 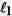 and 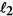 based methods.

	**DRO-DOT**	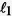 **-minimization**	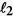 **-minimization**
AR	0.98	0.97	1.13
VR	0.97	1.05	1.24
CR	87.25	86.38	17.90
RT(s)	2.61	13.55	2.73

**Table 2:** Comparison of DRO-DOT reconstruction for different optode geometries.

	**SD-I**	**SD-II**	**SD-III**	**SD-IV**
VR	0.97	0.92	1.02	1.02
CR	87.25	67.26	47.90	45.10
