# Supplementary material for: Dimensionality Reduction Based Optimization Algorithm for Sparse 3-D Image Reconstruction in Diffuse Optical Tomography
Source: Sci Rep. 2016 Mar 4;6:22242. doi: 10.1038/srep22242 (PMC4778023; doi:10.1038/srep22242)
Supplement: Supplementary Information [file srep22242-s1.pdf]

Supplementary Information for

Dimensionality Reduction Based Optimization Algorithm for Sparse 3-D Image Reconstruction in Diffuse Optical Tomography

Tanmoy Bhowmik[1], Zhou Ye[2], Hanli Liu[2,*], and Soontorn Oraintara[1,3]

[1]University of Texas at Arlington, Department of Electrical Engineering, Arlington, TX 76019, USA
[2]University of Texas at Arlington, Department of Bioengineering, Arlington, TX 76019, USA
[3]Biomedical Engineering, Mahidol University, Salaya 73170, Thailand
[*]hanli@uta.edu

1 Setting the Correlation Threshold in Step-1

The effective correlation threshold τ_e was set to ensure that the relative approximation error $||\frac{||A^\sharp x^\sharp - Ax||}{||Ax||}$ remains below 5%. For each of the DOT geometries studied in the paper, a set of 100 random test images were generated. For each of this random test images, we calculated the relative approximation error, $E(i)$ for $i = \{1, 2, \ldots, 100\}$, as a function of correlation threshold τ. By the end of this process, we obtained 100 values of $E(i)$ for each value of τ and then averaged them to generate a single value of average relative approximation error, E_a for each value of τ. Finally, the effective correlation threshold τ_e was decided from the plot of average relative approximation error E_a vs correlation threshold τ by finding the point where E_a goes below 5%. Supplementary Fig. 1a shows such a plot for the DOT geometry of Fig. 1 in the paper. We also plot the dimensionality reduction factor $r = \frac{n-n^\sharp}{n}$ as a function of τ in Fig. 1b.

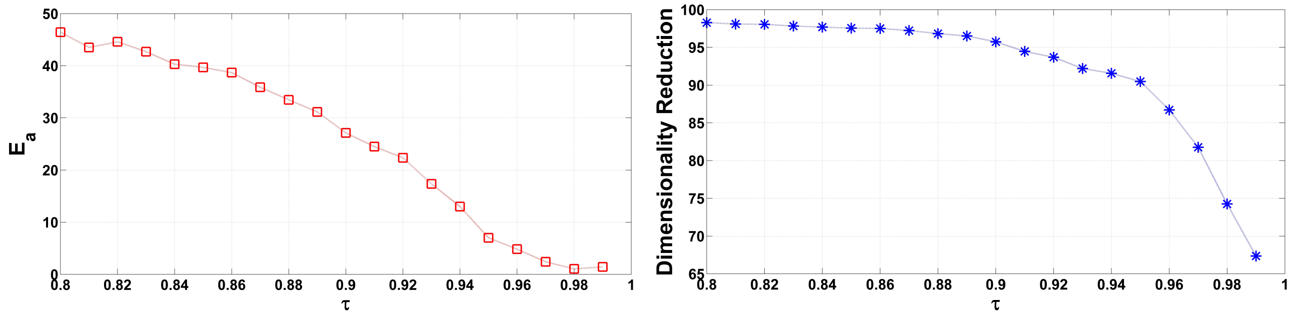

(a) (b)

Supplementary Figure. 1: Setting of τ_e in Step-1 and its effect on dimensionality reduction for the geometry of Fig. 1 in the paper. (a) Plot of average relative approximation error E_a vs correlation threshold τ and (b) Plot of % dimensionality reduction factor r vs correlation threshold τ. We set $\tau_e = 0.96$ as the effective correlation threshold where E_a goes below 5%. For the chosen value of τ_e, a reduction of dimensionality (or number of columns of A matrix) as much as 85% was achieved in step-1 of DRO-DOT

2 Convergence of SALSA

We plot the evolution of the cost function with time in Fig. 2 to illustrate the convergence of our algorithm. The convergence is shown for the phantom experiment in Fig. 1.

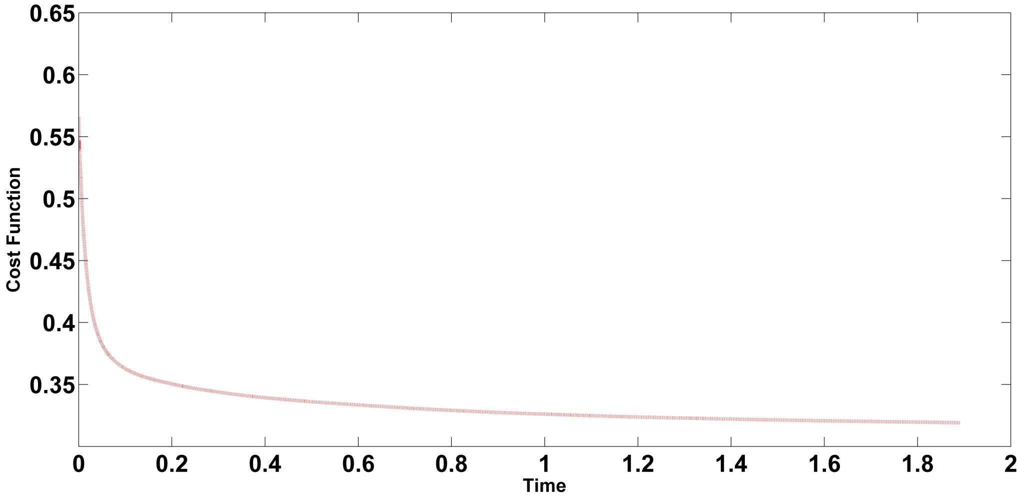

Supplementary Figure. 2: Convergence of SALSA in DRO-DOT step-1 for the phantom experiment in Fig. 1 of paper. The plot shows the evolution of the ℓ_1 regularized objective function with successive iteration time. SALSA coverges at $\simeq 1.9s$ i.e. at that time the relative error in the cost function betwen two successive iterations goes below our tolerance limit that we set as 10^{-5}.

3 Experimental Parameters

In our paper DRO-DOT algorithm has been applied for five different experimental set-ups: Four optode geometries for the brain imaging phantom study (Fig. 5) and one optode geometry for transcretal prostate phantom experiment (Fig. 7). In the table below we show the different parameters related to each of these experiment set-up:

Supplementary Table. 1: Parameters for different phantom experiments. Geometry 1-4 are for brain imaging phantom with different optode configuration as in Fig. 5 of the paper. Geometry 5 is for the transcretal prostate phantom whose geometry is shown in Fig. 7.

	Effetive Correlation Threshold (τ_e)	% dimensionality reduction r=$\frac{n-n^\sharp}{n}$	Regularization parameter (λ)	Time for convergence (step-1 and step-2)
Geometry 1	0.96	85	0.0251	1.90s and 0.17s
Geometry 2	0.95	88	0.0346	1.82s and 0.15s
Geometry 3	0.95	87	0.0371	1.85s and 0.16s
Geometry 4	0.97	81	0.0441	2.28s and 0.22s
Geometry5	0.96	85	0.0282	1.98s and 0.18s
